# Risk factors of emotional burnout in medical workers of the neurosurgery center: experience of Ukraine

**DOI:** 10.1192/j.eurpsy.2025.1989

**Published:** 2025-08-26

**Authors:** M. Markova, A. Regush, V. Pliekhov, A. Markov

**Affiliations:** 1 Sexology, Psychotherapy and Medical Psychology, Kharkiv National Medical University, Kharkiv; 2 Center of X-ray endovascular neurosurgery, Kyiv City Clinical Hospital № 1; 3 Psychology, Academy of Labour, Social Relations and Tourism, Kyiv, Ukraine

## Abstract

**Introduction:**

In the modern scientific discourse, there is a lack of data on risk factors for the occurrence of emotional burnout in employees of neurosurgical medical institutions. This is what determines the relevance of the study.

**Objectives:**

To determine the prevalence and risk factors of emotional burnout among medical workers of a neurosurgery center.

**Methods:**

In 2022-2024, a prospective study of the medical workers involved in emergency care for patients with acute ischemic stroke, in particular mechanical thrombectomy, was conducted at the Center for X-ray Endovascular Neurosurgery of the Kyiv City Clinical Hospital № 1. The average age of the sample was 32.2 ± 5.8 years. The gender structure was: 40 (80.0%) men and 10 (20.0%) women. The clinical-psychopathological, psychodiagnostic and statistical methods were used. The psychodiagnostic method was implemented by using the Maslach Burnout Inventory (MBI, C. Maslach et al., 1997).

**Results:**

It was determined that such a criterion as the gender of medical workers of the neurosurgery center does not affect the severity of any component of emotional burnout (p> 0.05). Statistically significant differences in the prevalence of high rates of emotional burnout components were determined between groups of medical workers with different specializations. In particular, it was determined that working in the center as a neurosurgeon is reliably associated with high rates for such components as “Emotional exhaustion” (p=0.04) and “Depersonalization” (p=0.006). A direct correlation was also established between the length of total work experience, length of service in the neurosurgery center and the number of working hours per month with the intensity of manifestations of emotional burnout in the selected contingent of individuals.

**Conclusions:**

The prevalence of emotional burnout among medical workers of the neurosurgery center has been established for various components of this phenomenon: “Emotional exhaustion” – 52.0%, “Depersonalization” – 40.0%, “Reduction of personal achievements”– 50.0%, which indicates a high level of stress, which negatively affects the mental health of the selected contingent. The risk of developing emotional burnout among the medical workers increases with increasing workload, length of service and length of service in the center. An absolute risk factor for emotional burnout is working as a neurosurgeon. The development of psychotherapeutic measures aimed at overcoming the manifestations of emotional burnout in medical workers and increasing their resilience to stressful working conditions of the neurosurgery center is promising.

**Disclosure of Interest:**

None Declared

